# 4′-Fluoro-2′-hydroxy­acetophenone

**DOI:** 10.1107/S1600536808011173

**Published:** 2008-04-26

**Authors:** Mohd. Razali Rizal, Seik Weng Ng

**Affiliations:** a Department of Chemistry, University of Malaya, 50603 Kuala Lumpur, Malaysia

## Abstract

The title compound, C_8_H_7_FO_2_, crystallizes as discrete mol­ecules, the conformation of which may be influenced by an intra­molecular hydr­oxy–carbonyl O—H⋯O hydrogen bond.

## Related literature

For the crystal structures of other substituted acetophenones, see: Filarowski *et al.* (2004 ▶, 2005 ▶); Hibbs *et al.* (2003 ▶); Huang *et al.* (2004 ▶); Ng (2007 ▶); Xu *et al.* (2005 ▶).
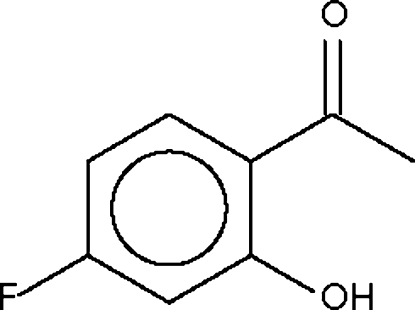


## Experimental

### 

#### Crystal data

C_8_H_7_FO_2_
*M* *_r_* = 154.14
Monoclinic, 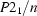

*a* = 3.7978 (1) Å
*b* = 14.2421 (3) Å
*c* = 13.0092 (3) Å
β = 91.884 (2)°
*V* = 703.27 (3) Å^3^
*Z* = 4
Mo *K*α radiation
μ = 0.12 mm^−1^
*T* = 100 (2) K
0.16 × 0.14 × 0.12 mm

#### Data collection

Bruker SMART APEX diffractometer
Absorption correction: none
8762 measured reflections
1601 independent reflections
1224 reflections with *I* > 2σ(*I*)
*R*_int_ = 0.039

#### Refinement

*R*[*F* ^2^ > 2σ(*F* ^2^)] = 0.043
*wR*(*F* ^2^) = 0.127
*S* = 1.05
1601 reflections
128 parameters
7 restraints
All H-atom parameters refined
Δρ_max_ = 0.30 e Å^−3^
Δρ_min_ = −0.28 e Å^−3^

### 

Data collection: *APEX2* (Bruker, 2007 ▶); cell refinement: *SAINT* (Bruker, 2007 ▶); data reduction: *SAINT*; program(s) used to solve structure: *SHELXS97* (Sheldrick, 2008 ▶); program(s) used to refine structure: *SHELXL97* (Sheldrick, 2008 ▶); molecular graphics: *X-SEED* (Barbour, 2001 ▶); software used to prepare material for publication: *publCIF* (Westrip, 2008 ▶).

## Supplementary Material

Crystal structure: contains datablocks global, I. DOI: 10.1107/S1600536808011173/lh2605sup1.cif

Structure factors: contains datablocks I. DOI: 10.1107/S1600536808011173/lh2605Isup2.hkl

Additional supplementary materials:  crystallographic information; 3D view; checkCIF report

## Figures and Tables

**Table 1:** Hydrogen-bond geometry (Å, °)

*D*—H⋯*A*	*D*—H	H⋯*A*	*D*⋯*A*	*D*—H⋯*A*
O1—H1⋯O2	0.857 (10)	1.76 (1)	2.554 (2)	154 (2)

**Fig. 1.**
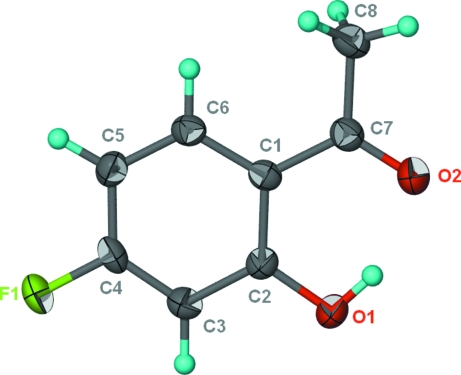
70% Probability thermal ellipsoid plot of 4'-fluoro-2'-hydroxyacetophenone. Hydrogen atoms are drawn as spheres of arbitrary radius.

## supplementary crystallographic information

### Comment

Acetopheonone is a liquid at room temperature. If a small substituent such as 5'-bromo (Ng, 2007), 5'-chloro (Filarowski *et al.*, 2004), 6'-hydroxy (Huang *et al.*, 2004), 5'-nitro (Hibbs *et al.*, 2003), 4'-methoxy (Filarowski *et al.*, 2005; Xu *et al.*, 2005) or 6'-methoxy (Filarowski *et al.*, 2005) is present the compounds exists as crystalline solids. The compound (I) containing the reltively smaller F substituent sublimes at room temperature. The structure contains discrete molecules (Fig. 1), in which the conformation may be influenced by an intramolecular hydrogen bond between the hydroxy and carbonyl groups.

### Experimental

The compound was purchased from Aldrich Chemical Company; the chemical exists as prismatic crystals.

### Refinement

All H-atoms were located in a difference Fourier map, and were refined with distance restraints of C—H 0.99±0.01 Å and O–H 0.84±0.01 Å. Their temperature factors were freely refined.

### Crystal data

**Table:**

C_8_H_7_FO_2_	*F*_000_ = 320
*M**_r_* = 154.14	*D*_x_ = 1.456 Mg m^−^^3^
Monoclinic, *P*2_1_/*n*	Mo *K*α radiation λ = 0.71073 Å
Hall symbol: -P 2yn	Cell parameters from 1854 reflections
*a* = 3.7978 (1) Å	θ = 2.9–26.4º
*b* = 14.2421 (3) Å	µ = 0.12 mm^−^^1^
*c* = 13.0092 (3) Å	*T* = 100 (2) K
β = 91.884 (2)º	Prism, colorless
*V* = 703.27 (3) Å^3^	0.16 × 0.14 × 0.12 mm
*Z* = 4	

### Data collection

**Table:**

Bruker SMART APEXII diffractometer	1224 reflections with *I* > 2σ(*I*)
Radiation source: fine-focus sealed tube	*R*_int_ = 0.039
Monochromator: graphite	θ_max_ = 27.5º
*T* = 100(2) K	θ_min_ = 2.1º
ω scans	*h* = −4→4
Absorption correction: none	*k* = −18→18
8762 measured reflections	*l* = −16→16
1601 independent reflections	

### Refinement

**Table:**

Refinement on *F*^2^	Secondary atom site location: difference Fourier map
Least-squares matrix: full	Hydrogen site location: inferred from neighbouring sites
*R*[*F*^2^ > 2σ(*F*^2^)] = 0.043	All H-atom parameters refined
*wR*(*F*^2^) = 0.127	*w* = 1/[σ^2^(*F*_o_^2^) + (0.0775*P*)^2^ + 0.0798*P*] where *P* = (*F*_o_^2^ + 2*F*_c_^2^)/3
*S* = 1.05	(Δ/σ)_max_ = 0.001
1601 reflections	Δρ_max_ = 0.30 e Å^−^^3^
128 parameters	Δρ_min_ = −0.28 e Å^−^^3^
7 restraints	Extinction correction: none
Primary atom site location: structure-invariant direct methods	

### Fractional atomic coordinates and isotropic or equivalent isotropic displacement parameters (Å^2^)

**Table:**

	*x*	*y*	*z*	*U*_iso_*/*U*_eq_	
F1	0.1828 (3)	0.50469 (6)	0.65495 (7)	0.0319 (3)	
O1	0.4466 (3)	0.72547 (8)	0.40329 (8)	0.0325 (3)	
O2	0.7055 (3)	0.88271 (8)	0.46250 (8)	0.0320 (3)	
C1	0.5542 (4)	0.76649 (10)	0.58170 (11)	0.0196 (3)	
C2	0.4380 (4)	0.70349 (10)	0.50349 (11)	0.0211 (3)	
C3	0.3106 (4)	0.61483 (10)	0.52868 (11)	0.0229 (3)	
C4	0.3051 (4)	0.59128 (10)	0.63045 (12)	0.0225 (4)	
C5	0.4165 (4)	0.64954 (10)	0.71015 (11)	0.0235 (4)	
C6	0.5398 (4)	0.73716 (10)	0.68410 (11)	0.0216 (4)	
C7	0.6909 (4)	0.85914 (10)	0.55377 (11)	0.0227 (4)	
C8	0.8132 (5)	0.92675 (11)	0.63595 (12)	0.0267 (4)	
H1	0.519 (6)	0.7824 (9)	0.4037 (19)	0.066 (8)*	
H3	0.221 (5)	0.5733 (10)	0.4729 (11)	0.029 (5)*	
H5	0.412 (5)	0.6279 (11)	0.7807 (8)	0.023 (4)*	
H6	0.616 (4)	0.7801 (10)	0.7398 (10)	0.024 (4)*	
H81	0.602 (4)	0.9492 (14)	0.6716 (15)	0.051 (6)*	
H82	0.968 (4)	0.8980 (12)	0.6899 (12)	0.033 (5)*	
H83	0.937 (5)	0.9790 (11)	0.6047 (15)	0.044 (5)*	

### Atomic displacement parameters (Å^2^)

**Table:**

	*U*^11^	*U*^22^	*U*^33^	*U*^12^	*U*^13^	*U*^23^
F1	0.0427 (6)	0.0200 (5)	0.0331 (5)	−0.0081 (4)	0.0043 (4)	0.0043 (4)
O1	0.0514 (8)	0.0275 (6)	0.0182 (6)	−0.0115 (6)	−0.0021 (5)	0.0018 (4)
O2	0.0457 (8)	0.0252 (6)	0.0254 (6)	−0.0091 (5)	0.0038 (5)	0.0027 (4)
C1	0.0191 (8)	0.0176 (7)	0.0221 (7)	0.0013 (5)	0.0012 (6)	−0.0019 (5)
C2	0.0223 (8)	0.0221 (8)	0.0187 (7)	−0.0003 (6)	0.0002 (6)	0.0003 (5)
C3	0.0235 (8)	0.0207 (7)	0.0244 (8)	−0.0012 (6)	−0.0003 (6)	−0.0023 (6)
C4	0.0227 (8)	0.0146 (7)	0.0304 (8)	−0.0005 (6)	0.0045 (6)	0.0030 (6)
C5	0.0266 (9)	0.0240 (8)	0.0201 (7)	0.0020 (6)	0.0028 (6)	0.0026 (6)
C6	0.0226 (8)	0.0205 (7)	0.0217 (7)	0.0022 (6)	0.0007 (6)	−0.0022 (5)
C7	0.0231 (8)	0.0204 (7)	0.0247 (8)	0.0006 (6)	0.0021 (6)	−0.0004 (6)
C8	0.0277 (9)	0.0220 (8)	0.0304 (8)	−0.0035 (6)	0.0026 (7)	−0.0032 (6)

### Geometric parameters (Å, °)

**Table:**

F1—C4	1.3594 (16)	C3—H3	0.988 (9)
O1—C2	1.3420 (17)	C4—C5	1.383 (2)
O1—H1	0.857 (10)	C5—C6	1.379 (2)
O2—C7	1.2370 (18)	C5—H5	0.969 (9)
C1—C6	1.399 (2)	C6—H6	0.984 (9)
C1—C2	1.416 (2)	C7—C8	1.501 (2)
C1—C7	1.468 (2)	C8—H81	0.993 (10)
C2—C3	1.395 (2)	C8—H82	0.989 (9)
C3—C4	1.367 (2)	C8—H83	0.977 (10)
			
C2—O1—H1	103.5 (17)	C6—C5—H5	122.5 (10)
C6—C1—C2	118.29 (13)	C4—C5—H5	120.3 (10)
C6—C1—C7	121.96 (13)	C5—C6—C1	121.90 (13)
C2—C1—C7	119.74 (13)	C5—C6—H6	118.4 (10)
O1—C2—C3	117.32 (13)	C1—C6—H6	119.7 (10)
O1—C2—C1	122.21 (13)	O2—C7—C1	120.59 (13)
C3—C2—C1	120.47 (13)	O2—C7—C8	119.14 (14)
C4—C3—C2	117.78 (14)	C1—C7—C8	120.27 (13)
C4—C3—H3	123.3 (11)	C7—C8—H81	107.6 (13)
C2—C3—H3	118.9 (10)	C7—C8—H82	113.7 (11)
F1—C4—C3	117.77 (13)	H81—C8—H82	105.8 (17)
F1—C4—C5	117.83 (13)	C7—C8—H83	109.5 (12)
C3—C4—C5	124.40 (14)	H81—C8—H83	111.0 (18)
C6—C5—C4	117.17 (13)	H82—C8—H83	109.2 (17)
			
C6—C1—C2—O1	−179.20 (14)	C3—C4—C5—C6	0.2 (2)
C7—C1—C2—O1	−0.3 (2)	C4—C5—C6—C1	−0.3 (2)
C6—C1—C2—C3	0.4 (2)	C2—C1—C6—C5	0.0 (2)
C7—C1—C2—C3	179.37 (14)	C7—C1—C6—C5	−178.94 (14)
O1—C2—C3—C4	179.15 (14)	C6—C1—C7—O2	178.92 (14)
C1—C2—C3—C4	−0.5 (2)	C2—C1—C7—O2	0.0 (2)
C2—C3—C4—F1	−179.64 (13)	C6—C1—C7—C8	−1.4 (2)
C2—C3—C4—C5	0.2 (2)	C2—C1—C7—C8	179.68 (14)
F1—C4—C5—C6	−179.97 (13)		

### Hydrogen-bond geometry (Å, °)

**Table:**

*D*—H···*A*	*D*—H	H···*A*	*D*···*A*	*D*—H···*A*
O1—H1···O2	0.857 (10)	1.76 (1)	2.554 (2)	154 (2)
